# The Spectrum of Coronary Artery Disease in Elite Endurance Athletes—A Long-Standing Debate: State-of-the-Art Review

**DOI:** 10.3390/jcm13175144

**Published:** 2024-08-30

**Authors:** Mihail Celeski, Giuseppe Di Gioia, Annunziata Nusca, Andrea Segreti, Maria Rosaria Squeo, Erika Lemme, Federica Mango, Armando Ferrera, Gian Paolo Ussia, Francesco Grigioni

**Affiliations:** 1 Fondazione Policlinico Universitario Campus Bio-Medico, Via Alvaro del Portillo, 200, 00128 Roma, Italy; mihail.celeski@unicampus.it (M.C.); a.nusca@policlinicocampus.it (A.N.); a.segreti@policlinicocampus.it (A.S.); g.ussia@policlinicocampus.it (G.P.U.); f.grigioni@policlinicocampus.it (F.G.); 2 Unit of Cardiovascular Sciences, Department of Medicine and Surgery, Università Campus Bio-Medico di Roma, Via Alvaro del Portillo, 21, 00128 Roma, Italy; 3 Institute of Sports Medicine and Science, National Italian Olympic Committee, Largo Piero Gabrielli, 1, 00197 Roma, Italy; mariarosaria.squeo@coni.it (M.R.S.); erikalemme@msn.com (E.L.); federicamango.md@gmail.com (F.M.); armando.ferrera95@gmail.com (A.F.); 4 Department of Movement, Human and Health Sciences, University of Rome “Foro Italico”, Piazza Lauro de Bosis, 6, 00135 Roma, Italy; 5 Clinical and Molecular Medicine Department, Sapienza University of Rome, 00198 Roma, Italy

**Keywords:** coronary artery disease, chronic coronary syndrome, elite athletes, endurance athletes, coronary plaque

## Abstract

Physical activity is recommended for the prevention of primary and secondary cardiovascular (CV) disease as it is linked to a number of health benefits, especially CV. However, recent research suggests that high-volume, long-term endurance exercise may hasten rather than slow the coronary atherosclerosis progression. This contentious theory has generated a great discussion and is still a major source of doubt when it comes to the clinical treatment of coronary artery disease (CAD) in athletes. CAD is the primary cause of sudden cardiac death in athletes over 35 years. Thus, recent studies evaluated the prevalence of CAD in athletes and its clinical and prognostic implications. Indeed, many studies have shown a relationship between endurance sports and higher volumes of coronary calcified plaque as determined by computed tomography. However, the precise pathogenetic substrate for the existence of an increased coronary calcification burden among endurance athletes remains unclear. Moreover, the idea that coronary plaques in elite athletes present a benign morphology has been cast into doubt by some recent studies showing potential association with adverse cardiovascular events. This review aims to analyze the association between physical activity and CAD, explaining possible underlying mechanisms of atherosclerotic progression and non-ischemic coronary lesions, focusing primarily on clinical and prognostic implications, multimodal evaluation, and management of CAD in endurance athletes.

## 1. Introduction

Exercise training and physical activity (PA) are crucial approaches for lowering the risk of cardiovascular (CV) events. Elite endurance athletes often surpass the recommended weekly threshold of 150 min of moderate exercise or 75 min of intense exercise by the current guidelines for CV prevention [1]. However, a reverse J-shaped dose–response association between cumulative exercise exposure and CV morbidity has been reported in multiple cases over the years, indicating that there may be a threshold beyond which there is a loss of some of the previously noted exercise-related advantages [2,3]. Indeed, the possible harmful effects of lifetime high-intensity endurance exercise on the heart are a topic of long-standing discussion [4]. 

In athletes older than 35, coronary artery disease (CAD) is the leading cause of sudden cardiac death (SCD), with clinical manifestations occurring in only 12–36% of the individuals [5,6]. Indeed, there is evidence that athletes are not immune to the formation of coronary artery plaques, which challenges our knowledge of the relationship between exercise and coronary health, emphasizing the relevance of a careful CV evaluation in athletes [7,8].

High-volume, high-intensity exercise training may actually raise the incidence and severity of subclinical coronary atherosclerosis, according to a number of recent research [9,10,11,12,13]. Notably, examination of the athletes’ plaque morphology revealed less mixed plaques and more frequently calcified plaques, indicating a more benign atherosclerotic pattern [9]. However, it is mostly unclear which is the underlying mechanism of atherosclerosis in elite athletes. Furthermore, it is still uncertain the clinical implications and the best management strategy for CAD in athletes.

Therefore, the aim of this narrative review is to explain current knowledge about the association between exercise and CAD, explaining potential underlying mechanisms of atherosclerosis and coronary plaque characteristics in athletes and focusing on their clinical and prognostic implications. There are also discussed other potential causes of exercise-induced ischemia and methods for the evaluation of athletes with suspected CAD, highlighting the challenges in the management of these patients.

## 2. Materials and Methods

We conducted a comprehensive literature search for data on articles published from 1990 to 2024 in PubMed, PMC, SportDiscus, and Cochrane reporting exercise-related issues related to coronary diseases. The main search terms were coronary artery disease in athletes, coronary plaque, coronary artery calcification in athletes, exercise-induced ischemia, endurance sports, coronary artery anomalies, myocardial bridge, and coronary artery dissection. To conduct further investigation, we additionally searched through the publications’ references that were collected. Clinical investigations, expert consensus, guidelines, case series, case reports, and narrative and systematic reviews constituted most of the articles that were considered. The search and result description were arranged into the following primary sections: exercise and coronary artery disease, a long-standing paradox; features of coronary atherosclerosis in endurance athletes; special subgroups of coronary anomalies and lesions in elite athletes; management of athletes with suspected coronary artery disease; conclusions.

## 3. Exercise and Coronary Artery Disease, a Long-Standing Paradox

### 3.1. Benefits of PA and Cardiac Adaptation or “Maladaptation”

PA is recommended for primary and secondary CV disease prevention and is linked to several beneficial effects. A mere 15 min of PA daily is linked to a 14% decrease in all mortality causes. Conversely, a population-attributable proportion of 12% of CV disease mortality is caused by inadequate PA. Since atherosclerotic disease accounts for more than 44% of CV deaths, exercise is consequently one of the best ways to lower the risk of major CV events [14,15]. Therefore, the current European Society of Cardiology (ESC) guidelines on sports cardiology and exercise in patients with CV disease recommend at least 150 min per week of moderate-intensity or 75 min per week of vigorous-intensity aerobic exercise in healthy individuals [16].

Exercise seems to be cardioprotective, partially driven by the positive impact on several atherosclerotic risk factors such as high blood pressure (BP), dyslipidemia, obesity, and diabetes [17]. However, it has advantageous benefits on the heart muscle and endothelial function as well. Molecular mediator generation, variations in the neurohormonal release, and maintaining oxidant/antioxidant equilibrium are only a few of the many underlying positive effects on the endothelium. Furthermore, regular exercise causes the vascular endothelium to exhibit anti-inflammatory properties and releases myokines from the skeletal muscle, enhancing vascular homeostasis and vasodilator capability through nitric oxide production [18,19]. Additionally, exercise promotes angiogenesis, enhancing oxygen delivery and decreasing adhesion molecules involved in the inflammatory atherosclerotic process [19].

Competitive athletes, who maintain lifetime extremely high levels of PA usually outlive sedentary and normally active individuals [20]. They also acquire advantageous adaptive CV characteristics such as increased myocardial and vascular compliance and heart remodeling [21,22]. Sports that need endurance are those that require long-term, intense, high-dynamic exercise, such as running or cycling, while strength training requires explosive muscle power, such as weightlifting. On the other hand, mixed sports, such as ball and team sports (like soccer or basketball), involve alternating stages of dynamic and/or static effort and recovery [23,24]. Static activity creates pressure stress on the left ventricle, whereas dynamic exercise largely applies a volume load. Training in sports with a high degree of dynamic component causes athletes to significantly increase their absolute left ventricular (LV) mass and chamber size (a condition known as eccentric hypertrophy). Conversely, with strength training, repeated bursts cause a considerable rise in blood pressure and heart rate, which increases LV mass but does not expand the size of the chamber (concentric hypertrophy) [23,24]. During competitions, there can be a significant increase in pulmonary artery pressure by up to 70% and important and constantly elevated cardiac output as a consequence of the elevated diastolic filling and ventricular emptying [25]. Despite these determinants, power output also plays a crucial role in determining the performance of athletes across various sports during competition [26]. Moreover, elite athletes have less important ventricular dilatation, but greater left atrial size and LV hypertrophy as compared to young athletes. However, LV ejection fraction (LVEF) remains preserved from a functional standpoint, but the stroke volume and diastolic function parameters increase [9]. 

Nonetheless, there is ongoing discussion on the health status of those who significantly exceed the minimum recommendations. Indeed, in the case of excessive exercise, defined as more than 150 min per week of moderate-intensity or 75 min per week of vigorous-intensity aerobic exercise, there is a potential for the CV system to become “maladapted”, which would put athletes at higher risk of CV and all-cause mortality [27]. Whether physically demanding sports activities have a negative impact on the heart over time is a long-standing debate. According to one theory, there is a reverse J-shaped relationship between the effects on the CV system and the intensity/frequency of PA [28]. It is suggested that although moderate exercise would be beneficial in comparison to a sedentary lifestyle, extremely intense PA would be detrimental to CV health [28,29]. Indeed, excessive exercise volumes have been linked to cardiac dysfunctional adaptation leading to atrial fibrillation, myocardial fibrosis, exercise-induced cardiac biomarker release, and accelerated coronary artery calcification. Furthermore, excessive PA may raise the risk of myocardial infarction (MI) and SCD, particularly in cases where it is undertaken by untrained individuals [30]. Therefore, athletes are not exempt from CAD. Unexpectedly, new research suggests that high-volume, long-term endurance exercise could accelerate rather than slow the progression of coronary atherosclerosis [9,10,11,12,13]. This causes controversy because the primary cause of exercise-related SCD in elite athletes is atherosclerosis [31]. Plaque rupture and its consequences, including demand ischemia resulting in ventricular arrhythmia or ventricular arrhythmia from a previous scar, are the mechanisms of SCD in elite athletes with underlying CAD [32]. However, the underlying mechanisms of atherosclerosis and plaque formation in athletes remain still unclear.

### 3.2. Coronary Artery Disease in Elite Athletes

There is currently enough data to suggest the existence of a minor but significant risk of atherosclerotic CAD among endurance athletes despite the well-established and indisputable health advantages of regular physical training. Indeed, it has been shown that there is a paradoxical relationship between long-term marathon running involvement and increasing calcified coronary plaque volume as determined by computed tomography coronary angiography (CCTA). Even in athletes with low atherosclerotic risk profiles, as determined by the widely used risk score algorithms, this connection appears to exist regardless of the existence of CV risk factors [10].

There is growing evidence showing that male elite athletes, when compared to age and atherosclerotic risk-matched controls, have higher coronary artery calcium (CAC) scores and a higher prevalence of coronary atherosclerosis on CCTA [9,10]. For example, in the Measuring Athlete’s Risk of Cardiovascular Events (MARC) study, 318 middle-aged male athletes who could exercise to high workloads (318 ± 48 Watts) were included, most of them having a low European Society of Cardiology Systematic Coronary Risk Evaluation (SCORE) risk. In 60 (16%) of them, there was an occult CAD defined as CAC > 100 or coronary narrowing by more than 50% on CCTA in athletes with CAC < 100 [33]. Another important study on CAD is the one conducted by Merghani et which enrolled 152 competitive runners and cyclists (age = 55 ± 9 years) and 92 age-matched, normally active controls [9]. The controls did not have a family history of premature CAD (<40 years), a prior diagnosis of CAD, or other common CV risk factors. Compared to sedentary individuals, male athletes had a higher incidence of atherosclerotic plaques of any luminal irregularity (44.3% vs. 22.2%; *p* = 0.009), and only male athletes had luminal stenosis of at least 50% (7.5%) and a CAC > 300 (11.3%). In addition, controls had mixed-morphology coronary plaques, while male athletes had mostly calcified plaques. This suggested that distinct pathophysiological mechanisms may be at play for the development of plaque in sedentary athletes [9].

To better understand how common subclinical atherosclerosis is connected to CV risk factors and how it affects myocardial damage and outcomes, Mohlenkamp et al. conducted a study enrolling experienced recreational marathon runners. There were 108 apparently healthy individuals who were 50 years of age or older, had run at least five full-length marathons (42.195 km) in the three years before, and had no history of established cardiac disease, compared to age- and Framingham risk score (FRS)-matched controls. Despite the significantly lower FRS, 36% of the runners had a CAC score (CACS) > 100, which was comparable to age-matched controls. However, in contrast to FRS-matched controls, marathon runners had greater rates of CAC (median CAC: 36 vs. 12, *p* = 0.02) [10].

In addition, Tsiflikas et al. also examined male marathoners over 45 years of age and found that half of them had CAD, with 24% having plaques in the proximal coronary segments [34]. However, the connection between activity and CACS is still up for debate. In the Boston MASTER study, previous tobacco use and a family history of early atherosclerosis disease were shown to be the most common concerns among approximately 65% of elite athletes who had at least one documented CV risk factor. They demonstrated that there was little correlation between CAD and any aspect of previous exercise exposure and that CAD seemed to be connected with classic atherosclerotic risk factors, such as dyslipidemia and hypertension [35]. 

On the contrary, DeFina et al. examined 21,758 men and discovered that, in comparison to less physically active groups, extremely active men with >3000 metabolic equivalent of task (MET)-min·wk^−1^ of activity had an 11% greater risk of CAC > 100 AU [13]. Furthermore, 284 middle-aged males who played competitive or recreational sports in a different study conducted by Aengevaeren et al. had CCTA examinations and CAC measurements. To determine MET minutes per week, exercise volumes were multiplied with MET ratings. The movement of the participants was classified as being <1000, 1000 to 2000, or >2000 MET-min/week. The authors conclude that high levels of exercise are linked to an increased incidence of atherosclerotic and CAC plaque, as well as a propensity for calcified rather than mixed plaque [11]. These results were considered benign and non-alarming since calcified plaques are less likely to rupture and more stable than mixed and non-calcified plaques [36]. 

Nevertheless, the clinical and prognostic implications remain still speculative, as discussed later.

### 3.3. Clinical and Prognostic Implications—A Long-Standing Debate

Whether a high CACS and subclinical CAD in athletes have detrimental clinical and prognostic effects is still a controversy. 

Athletes who engage in lifelong, high-intensity PA may have a worse prognosis. Indeed, nearly 900 sudden sports-related deaths were recorded in a prospective national study carried out in France between 2005 and 2010, the majority of which (95%) were caused by CAD and largely involved young male athletes [5]. Athletes with pre-existing CAD may be more susceptible to myocardial infarction during periods of strenuous PA. Therefore, the focus of these patients’ therapy should be on assessing their risk of inducible ischemia. It has been demonstrated before that although athletes with raised CACS typically have calcified plaque morphology, which lowers risk, it is widely known that those with elevated CACS have a much higher risk of severe cardiac events compared to those with a CACS of 0. In fact, a CACS of 400 is linked to an estimated 34% chance of major adverse cardiac events (MACE), whereas a CACS of 0 relates to a 2.1% risk. In particular, those with CACS > 1000 seem to be at extremely high risk of both CV disease and all-cause death [37]. In accordance, zero CACS was less common in the Mohlenkamp et al. trial than it was in the MARC study (28.7% vs. 47.5%, *p* < 0.001). Additionally, it was revealed that CV events almost entirely occurred in those with a CACS greater than 100 AU, which was more common than in MARC (36.1% vs. 16.4%, *p* < 0.001) [10,33]. 

A very important prognostic piece of information was provided by the previously reported study by Mohlenkamp et al. In their study, the presence of myocardial injury was independently predicted by CAC percentile levels, as demonstrated by the fact that 12% of the runners had ischemic-type late-gadolinium enhancement (LGE) on the cardiac magnetic resonance (CMR). Follow-up data on CV events (21 months) were also included in the study. Out of the 38 athletes with CAC > 100, 4 athletes had coronary events (2 aborted cardiac arrests after PA, 1 acute myocardial infarction, and 1 surgical revascularization for CAD revealed by additional testing), and 3 of these athletes had LGE. However, there were no cardiac deaths reported in the study. These findings imply that subendocardial fibrosis in elite athletes could be a sign of micro-emboli, coronary spasm, and subclinical myocardial infarction due to demand ischemia. Remarkably, no participant with CAC = 0 experienced events. In contrast, cardiac events occurred in 8% and 14% of the athletes with CAC 100–400 and >400, respectively [10]. However, the study’s cross-sectional study methodology works well for identifying correlations but is not appropriate for determining causal links between exposures and clinical characteristics. While it is plausible to conclude that aging endurance athletes would have remarkably high levels of CAC, the underlying mechanisms are still unknown. Although inviting information, drawing the premature conclusion that intense exercise is the key mechanistic factor causing CAC is still premature. These results do not advocate for a decrease in PA because a lower level of exercise can be linked to the advancement of atherosclerosis. In fact, in 2013, Delaney and colleagues classified Multi-Ethnic Study of Atherosclerosis (MESA) study participants who underwent a follow-up CCTA scan based on their level of PA and showing that a lower level of PA was found to be highly associated with the progression of coronary calcification in athletes who already had it at baseline [38].

On the contrary, other research suggested that physical exercise is not linked to an increased risk of CV events, even in the presence of a high CACS. Indeed, Gao et al. supported earlier research showing a link between the general population’s elevated risk of CV events and the advancement of CAC. However, they noted that the risk was constant regardless of PA level [39]. Nevertheless, other studies have demonstrated that PA is not linked to an increased risk of CV death or events, even in athletes with high CAC scores. Thus, in 2022, German et al. verified these findings by classifying the 6000 participants in the MESA trial into two risk groups based on their levels of PA and CAC: low-risk (CAC < 100) and high-risk (CAC > 100) subjects. Their findings demonstrated that an increase in PA was associated with a decreased risk of all-cause mortality [40]. Moreover, in a cohort of 8000 subjects, Redford et al. showed that higher cardiorespiratory fitness decreased CAD risk, whereas higher CAC increased it. When considered collectively, as CAC levels grew, an increase in training level and, by extension, cardiorespiratory fitness (CRF) reduced the risk of CV disease events after adjusting for CACS. The incident rate decreased by 14% with each MET increase in CRF across CAC scores that had been adjusted for risk variables [12].

The lack of PA impact on CV outcomes was recently confirmed by another trial. In DeFina et al.’s study, described previously, athletes were followed up for ten years. The researchers discovered that, among men with CAC > 100 AU, those with >3000 MET-min·wk^−1^ of activity did not show increased CV or all-cause mortality when compared to those with less than 1500 MET-min·wk^−1^. This suggests that high levels of PA did not increase mortality when CAC was elevated [13]. The lack of women in this registry, however, was an important study limit.

To explain why highly trained endurance athletes have a lower risk of CV events compared to non-athletes, there is a theory that their plaque composition is more benign. Given their decreased risk of rupture, athletes with calcified plaques are thought to have relatively benign plaque formation. In contrast to sedentary members of the general population, endurance athletes have higher volumes and prevalence of coronary plaques. However, the benign nature of these high-density plaques might indicate a better CV prognosis, as demonstrated by Aengevaeren et al. in their previously reported study [11].

However, there is some contradiction associated with the higher frequency of atherosclerotic plaque, the lower incidence of CV disease, and longer lifespans among professional athletes. Indeed, given the lack of ability to assess temporal correlations, these observations must be taken into account in light of the inherent limitations of cross-sectional investigations. Another limit that may be mentioned is the athletes’ overall atherosclerotic burden, reflected by the CACS, which may be overestimated if exercise increases the degree and density of endothelium calcification. If this is the case, the CACS may not be as reliable in predicting CV morbidity and mortality risk in athletes as it is in the general population when used as a surrogate marker to quantify coronary atherosclerotic volume. Furthermore, determining the predictive importance of subclinical atherosclerosis among athletes is difficult due to the low incidence rate of CV outcomes in many study populations. The phenomena of increased plaque burden but decreased risk of adverse CV events in endurance athletes may be better understood by separating analyses that look at the risk of rupture from that of plaque progression. Another possible reason for the lack of evidence showing a rise in unfavorable consequences among athletes with CAC could be that long-term endurance athletes acquire a variety of preventive CV adaptations that lead to a considerable increase in coronary flow reserve. These consist of coronary collateralization, ischemic preconditioning, and optimum vasodilatory capacity as a result of increased nitrous oxide production. 

Despite this, the benign character of the athletes’ coronary atherosclerosis was called into question by another study. A balanced prospective observational investigation, the Master@Heart study, examined the absolute prevalence of various forms of coronary plaque in 191 male lifetime elite endurance athletes and 176 healthy non-athletes with low CV risk profiles [41]. In contrast to the other research, the Master@Heart study did not include any participants who had a history of smoking, dyslipidemia, arterial hypertension, or diabetes mellitus. Lifelong athletes had a larger overall burden of coronary plaque, with calcified plaques being the most common type in both athletes and non-athletes, followed by mixed and non-calcified plaques. However, a higher percentage of professional athletes had non-calcified and mixed-morphology plaques, proximal plaques, and lesions with considerable stenosis (OR 1.96, 95% CI 1.24–3.11) [41]. In fact, it is well known that the proximal position of coronary plaques, a stenosis grade of ≥50%, and non-calcified/mixed features are all known risk factors for ischemic heart disease [42,43]. The authors conclude that, compared to fit and healthy people with a similarly low CV risk profile, lifelong athletes do not have benign plaques but a higher rate of risk-prone non-calcified plaques in proximal segments. 

It is true that in the general population, CAC is linked to a higher risk of adverse cardiac events. However, elite athletes may not experience the same risks. Therefore, simply knowing one’s CAC should not discourage someone from increasing their PA levels. It is crucial to emphasize that not every coronary plaque is calcified and that calcification score is not a sign of unstable plaque [41]. Indeed, it is still vitally necessary to integrate CT findings with the identification of CV risk factors, ECG findings, and symptoms that may be underdiagnosed in athletes. 

This will remain an ongoing challenging debate until additional large-scale randomized long-term trials yield different findings.

## 4. Features of Coronary Atherosclerosis in Endurance Athletes

### 4.1. Mechanisms of Atherosclerotic Progression

The development of athletes’ atherosclerotic CAD has been suggested to be caused by multiple factors. The precise mechanisms, however, underlying the association between PA and the development of CAD, including coronary plaque calcification in athletes, are still uncertain. One explanation could be that elevated shear stress during exercise is caused by hyperdynamic blood flow in the coronary, which can lead to persistent endothelial damage. Exercise increases the wall stress along the coronary, especially at locations where turbulent blood flow occurs, such as bifurcations. As a consequence of the intensive exercise-related oscillatory flow dynamics, there is an endothelial dysfunction and formation of precursors known as fatty streaks, leading to atherosclerotic formation and progression [44,45]. The process of repairing damaged coronary endothelium results in elevated calcium deposition and the development of atherosclerotic plaque. Because of the severe repetitive calcification, this repetitive injury-related calcified plaque development stabilizes over time [46]. In these circumstances, athletes are often prescribed lipid-lowering therapy such as statins. However, statins, independently of their effects on plaque regression, increase the calcification of coronary atheroma. Indeed, the results of a recent study shed light on how statins may maintain plaque in addition to their ability to reduce plaque regression [47].

Even though it is plausible that the hemodynamic and mechanical elements of endurance training could cause artery wall damage and recovery calcification, this theory is still a hypothesis generated by some studies [9,11]. However, even though traditional atherosclerotic risk factors were attempted to be controlled in such studies, additional explanatory factors, such as dietary intake, psychological stress, use of anti-inflammatory medications, and chronic inflammation, as well as underlying atherosclerotic genetics were not adequately taken into account [9,11]. According to some other research, high levels of intensive exercise may actually accelerate the progression of pre-existing CAD rather than causing atherosclerotic plaque formation [48]. Moreover, during endurance training, increased mechanical pressures on calcified plaques may cause erosion or rupture of the plaque, which could lead to the production of epicardial thrombus and microembolization [49]. 

Despite the long-discussed mechanical factors, also the genetic predisposition, the presence of established CV risk factors, as well as an increase of parathyroid hormone (PTH) and acute pro-inflammatory state generation during intense exercise, can explain the atherosclerotic process in athletes [50]. Indeed, one of the main regulators of calcium homeostasis, PTH, is hypothesized to be linked to the pathogenesis of CV disease. On the other hand, as numerous in vivo and in vitro investigations have shown, exercise causes an elevation in serum PTH, whose levels are proportionally correlated with the duration and intensity of exercise [51,52]. Nevertheless, it is still unclear how exercise-induced increase of PTH affects the coronary vasculature directly.

Additionally, recent research explains that there is a role of inflammation in the development of atherosclerosis beyond the CV risk factors. Indeed, increased systemic release of pro-inflammatory cytokines and markers in this setting can be a consequence of intense exercise [48]. Furthermore, there is a strong correlation between the development and instability of atherosclerotic plaque and inflammatory mediators [53]. Nevertheless, after the excessive generation of inflammatory cytokines that accompanies endurance training, increasing vascular oxidative stress is likely to drive the atherosclerotic process even further. Regular exercising may have antioxidant benefits, while intense exercise sessions may suddenly increase vascular oxidative stress by producing oxygen radicals. Furthermore, as they encourage osteogenic development and calcium mineralization in vascular cells, oxygen radicals might be responsible for the connection to coronary artery calcification [54,55]. However, more research is needed to determine how these inflammatory pathways impact CAC in athletes.

**Figure 1:**
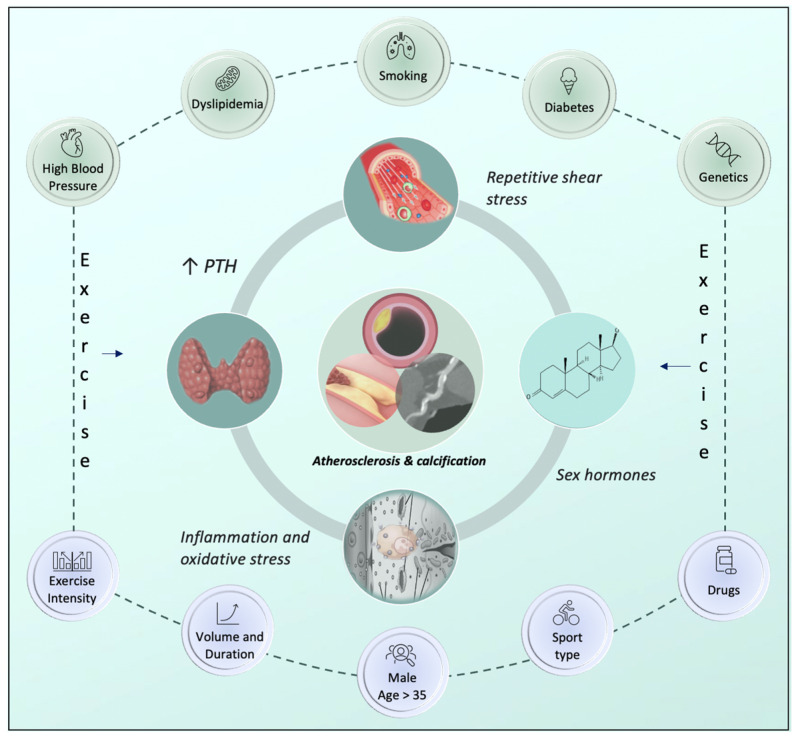
Mechanisms of exercise-related coronary atherosclerosis in athletes and contributing factors. PTH = parathyroid hormone.

In addition, a discussion about the role of sex hormones in atherosclerosis was raised by the higher occurrence of CAC among male endurance athletes. It has been noted that testosterone administration in experimental mice has both pro- and anti-calcific effects. There may be a specific threshold level of testosterone required to preserve CV health, as demonstrated by the calcification of vascular smooth muscle cells (VSMCs) induced by testosterone via the androgen receptor in one trial and the inhibition of VSMC calcification in a different one [56]. However, further research is required to determine testosterone’s contribution to atherosclerotic calcification. The main mechanisms of atherosclerosis formation in athletes are illustrated in Figure 1.

Beyond the different roles of the above-mentioned exercise-related potential factors, it is important to keep in mind that CAD in athletes is a complex and multifactorial process. Clarifying the pathophysiological mechanisms of atherosclerosis and coronary calcification brought on by prolonged, intense endurance exercise requires more investigation.

### 4.2. Coronary Artery Calcification and Plaque Characteristics in Athletes

Literature-based data have demonstrated that athletes’ plaques have comparatively distinct structures and that athletes are more likely to have calcified plaques than sedentary people, which are characterized mostly by a mixed plaque composition [11]. Indeed, the related CV risk is significantly impacted by the distinction of plaques into calcified, non-calcified, and mixed plaques, depending on their morphology. For instance, in a recent study, calcified plaques were linked to a 3-year MACE of 5.5% compared to 22.7% for non-calcified plaques and 37.7% for mixed plaques that are more prone to rupture [36]. The negative relationship between CV events and calcified plaques implies that despite the possibility of athletes having a higher prevalence of calcified coronary plaques, this may be a sort of adaptation that reduces their vulnerability to acute rupture [57]. 

As previously reported, endurance athletes have more frequently calcified plaques detected by CCTA and higher CAC values [58]. Indeed, a popular and effective method for classifying CV risk, forecasting patient outcomes, and directing preventive measures is CAC scoring. The two main CT techniques for measuring CAC are multi-detector computed tomography (MDCT) and electron-beam computed tomography (EBCT). One well-known measure of the overall burden of coronary atherosclerosis is the Agatston score, which is also the most extensively verified and commonly used score. It is calculated in Hounsfield units (HU) by combining the product of the entire plaque area and a cofactor determined by the plaque calcium attenuation [59,60]. The area of the lesion (Ai) multiplied by a weighting factor (wi) based on the maximal CT number (CTmax) in the region of interest yields the calcium score for each region of interest (CSi): Where wi is 1 if 130 HU CTmax < 200 HU, 2 if 200 HU ≤ CTmax < 300 HU, 3 if 300 HU ≤ CTmax < 400 HU, 4 if 400 HU ≤ CTmax. Total calcium score, also known as Agatston scores for individual arteries, individual calcifications, or the entire heart, is computed by adding the corresponding values for the regions of interest. Total plaque burden should be replaced by the total Agatston calcium score and the corresponding risk group, as follows: minimal for calcium score values from 0 to 9, mild for 10 to 99, moderate for 100 to 399, and severe for 400 or higher [61]. Therefore, beyond the scope of approved risk assessment instruments, numerous studies that have assessed the predictive usefulness of the CACS for incident CAD have consistently shown a notable improvement in risk discrimination and risk-reclassification indices [62,63]. While low scores do not rule out obstructive and high-risk plaques, a zero CAC score has a high negative predictive value for ruling out severe coronary atherosclerotic disease. On the other hand, patients with CAC > 100 have a risk similar to that of patients who have had prior CAD [64].

However, it is now widely understood that calcification in the coronary artery is an active pathogenic process, with ectopic bone production being the base of the process [65]. Indeed, the impact of calcification on plaque “stability” is a topic of ongoing debate, and some recent notions may cast into doubt the benign nature of CAD in athletes. Plaque stability is significantly influenced by the location, form, density, and size of calcium deposits, among other characteristics. Therefore, it is believed that plaques exhibiting a “spotty” or “speckled” calcification pattern, with tiny calcium deposits measuring approximately 500 μm in diameter, have a higher risk of rupture [8]. 

Even though early calcium deposition in the coronary vascular wall’s intimal layer might not be large enough to be seen on CCTA, with time, when these deposits expand and/or combine with nearby deposits, they will become noticeable. Moreover, the overall surface area of this interface is positively correlated with the quantity of concentrated stress. Rupture stress concentrates in the interface area between a stiff calcium deposit and the flexible surrounding tissue [66]. Thus, the total surface area will be higher if there are many little calcified deposits (like those shown in the speckled pattern), and the interface surface area may decrease when these deposits expand and merge into larger calcified tissue. Larger calcified deposits that expand and clump together, along with collagen deposition, appear to reduce the likelihood of plaque rupture near the surface [66,67]. Therefore, the inclusion of other imaging modalities or analysis may be helpful in improving risk stratification by offering a deeper assessment of plaque composition and related vulnerability, even though the CACS alone may reasonably predict CV events in the general population.

The necrotic core, fibrous cap, calcium, and inflammatory activity are the constituents of atherosclerosis. However, the definition of the plaque’s stability goes beyond just its level of calcification. Thus, endurance athletes’ increased prevalence of calcified plaques does not indicate that they are immune to CV events. Actually, a thin fibrous cap, big necrotic core, positive remodeling (remodeling index > 1.1), perivascular inflammation, and spotty calcifications are the histological characteristics that characterize a vulnerable plaque [68]. Furthermore, a particular high-risk attenuation pattern of atherosclerotic plaques on CCTA images is the “napkin ring sign” (defined as a low-CCTA-attenuated plaque core around a higher-CCTA-attenuated rim-like region), which has been documented in recent research [68]. Moreover, a recent study showed that participating in lifetime endurance sports is not necessarily linked to a more favorable coronary plaque composition as compared to leading a healthy lifestyle. More coronary plaques, including non-calcified plaques in proximal segments, were seen in lifetime endurance athletes compared to fit and healthy people with similar low CV risk profiles [41].

Therefore, it is important to remember that calcified plaques are not necessarily linked to a lower risk of CV events in athletes, and other characteristics that may define the stability of the plaque using intravascular imaging should be evaluated and tested in large-scale trials. 

### 4.3. Factors Impacting on Atherosclerotic Burden and Coronary Calcification

Numerous factors, including sex, intensity, race, and sport type, are yet unknown in terms of their impact on the development of CAD in athletes.

Women are significantly underrepresented in the literature regarding how high-dose exercise affects CV outcomes. There is a lack of data in female cohorts, which suggests that CAD is not very common. Mergani et al. studied nearly 240 individuals (controls and athletes) with low 10-year FRS for CAD, finding that male athletes had a higher probability of moderately to severely increased coronary CACS ≥ 300 and atherosclerotic plaques with any luminal abnormality than did controls. Yet, neither the number of plaques (15% vs. 21%, *p* = 0.57) nor the CAC ≥ 100 (7% vs. 11%, *p* = 0.62) nor the incidence of plaques differed between female athletes and their less active controls [9]. Additionally, compared to controls, research by Roberts et al. of 26 lifetime female marathon runners who had completed at least one marathon a year for 10–25 years revealed a reduced calcium burden, lower prevalence, and smaller volume of coronary artery plaque. The validity of this comparison is called into doubt by the fact that the athletes also had a reduced profile of atherosclerotic risk factors, whereas the control patients had a much greater body mass index and a higher prevalence of traditional CV risk factors [69].

Some research suggests that the amount and intensity of exercise may influence the course of atherosclerosis. In the Aengevaeren et al. study, when compared to persons with low lifelong activity volume (<1000 MET minutes per week), athletes with high lifelong exercise volume (>2000 MET minutes per week) had a higher CACS, CAC area, and nearly a 3-fold higher CAC and plaque prevalence [11]. Very strenuous activity (≥9 METs) and a high lifetime exercise volume were independently linked to the prevalence of coronary plaque and CAC. It is interesting to note that when compared to individuals in the low lifelong exercise volume (<1000 MET minutes per week) group, participants with prevalent atherosclerotic plaque on CCTA who had a high lifelong exercise volume had a lower prevalence of mixed plaque and more often had only calcified plaque [11]. Similar findings were seen in the MARC 2 trial, which followed middle-aged and older male athletes for six years. During that time, exercise intensity, but not volume, was linked to the progression of coronary atherosclerosis. The percentage of very vigorous-intensity exercise (≥9 MET hours/week) was linked to a higher progression of atherosclerotic plaques and coronary artery calcification, while the percentage of vigorous-intensity exercise (6 to 9 MET hours/week) was linked to a slower progression of coronary artery calcification. Although the finding’s clinical significance is still unknown, it could affect the CV risk related to coronary atherosclerosis [11]. On the other hand, Merghani et al., in their study, did not demonstrate any significant association between exercise dose and CAC > 70th percentile [9].

Moreover, without considering exercise features, it is difficult to distinguish the atherosclerotic burden based on the type of sport. While the majority of athletes in the studies by Merghani et al. and Molhenkamp et al. were runners, the athletic population in the trial by Aengevaeren et al. was more diversified, consisting of 25% runners and 29% cyclists [9,10,11]. Research indicates that runners are more likely than cyclists to develop coronary plaques [70]. Although cyclists tended to have more calcified plaques than runners, they had generally lower rates of coronary atherosclerosis. This finding could be explained by variations in exercise intensity or in the amounts of skeletal stress, bone turnover, and calcification that occur throughout the two sports [70]. However, cyclists made up the majority of the athlete population in the Master@Heart trial, where endurance training seemed to be associated with more non-calcified plaques in proximal segments, while runners made up the majority of the active control group [41]. The comparatively small sample size of subgroups could be one reason for the different results among studies. Furthermore, athletes may participate in multiple sports, making it challenging and unclear to assess the effects of a single sport. To definitively address the differences connected to sport, larger-scale future studies are required.

At last, it is known that race may affect CAC [71]. Nevertheless, most of the research has solely examined white people. Using race-specific analyses, Laddu et al. discovered distinct relationships between PA and CAC in African–American and white participants, indicating that race could influence this relationship. Indeed, they revealed that white but not African–American participants who were exceeding PA guidelines had significantly increased odds of CAC [72]. However, to address the racial disparities in CAD among athletes, more research is needed.

## 5. Special Subgroups of Coronary Anomalies and Lesions in Elite Athletes

**Figure 2:**
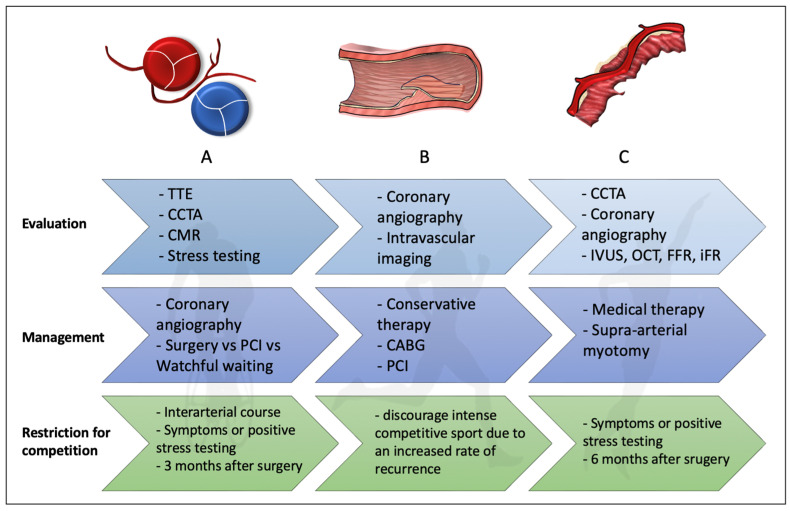
Special subgroups of coronary anomalies and lesions in elite athletes. (**A**) coronary artery anomalies (**B**) Spontaneous coronary artery dissection (**C**) Myocardial bridging. IVUS = intravascular ultrasound, CABG = coronary artery bypass graft, CCTA = coronary computed tomography angiography, CMR = cardiac magnetic resonance, FFR = fractional flow reserve, iFR = instantaneous wave-free ratio, OCT = optical coherence tomography, PCI = percutaneous coronary intervention, TTE = transthoracic echocardiography.

Apart from atherosclerotic CAD, myocardial ischemia and perhaps SCD are also linked to other entities, such as an anomalous origin of a coronary artery, myocardial bridge (MB), and spontaneous coronary artery dissection (SCAD) [73,74,75], represented in Figure 2. 

### 5.1. Coronary Artery Anomalies

An additional major cause of SCD in athletes is the abnormal origins of the coronary artery (AOCA). The left coronary artery (LCA) that arises from the right sinus of Valsalva, the right coronary artery (RCA) that arises from the left sinus of Valsalva, and those with an aberrant origin from the pulmonary artery are examples of coronary anomalies. Although exact estimates range from 0.5 to 1%, the occurrence in the general population is unknown [76].

As resting and stress ECG are often normal, anomalous coronary arteries are one of the most challenging disorders to identify in their preclinical stage [76]. In certain instances, the initial and distinct manifestation is regrettably an aborted case of SCD. Research indicates that a smaller percentage of athletes suffering from a solitary coronary artery anomaly exhibited early warning signs such as syncope or chest pain during exertion [77]. However, a coronary anomaly is mostly unintentionally found during a CCTA or transthoracic echocardiography (TTE) evaluation due to a different cause, like a heart murmur or an irregular ECG. 

It seems that aberrant arterial development or location causes ischemia alterations that lead to SCD. For instance, as an abnormal LCA passes between the aorta and the pulmonary arteries during exercise, it may be compressed by its intramural path within the aortic wall, resulting in repeated episodes of temporary ischemia [77]. These episodes might encourage cardiac fibrosis, which might place people at risk of ventricular arrhythmias. In athletes who have abnormal rest and/or stress test ECG, as well as symptoms such as palpitations, vertigo, dyspnea, chest pain, or discomfort during exertion, there should be a more detailed investigation [77]. In this particular scenario, the sports physicians ought to specifically request that the ostia and initial tracts of the coronary arteries be searched, and TTE should be carried out by physicians who possess specialized training and experience in identifying coronary arteries [78]. If an athlete has unexplained CV symptoms and their coronary origin cannot be seen, CMR or CCTA may be an option [77,79]. Additionally, high take-off can be present even though these types of coronary artery anomalies are uncommon. They are identified by TTE and are characterized by an origin above or distal to the sinu–tubular junction. They can occur alone or in conjunction with other cardiac abnormalities, and they typically involve RCA (up to 85% of cases) [80].

Following an athlete’s diagnosis of an AOCA, a cardiac catheterization should be done. A few highly qualified authors suggest that, as part of this process, an intravascular ultrasound examination of the anomalous artery should be done on a regular basis to provide a more accurate risk assessment. Evaluation of the instantaneous wave-free ratio (iFR) or fractional flow reserve (FFR) in the cath lab (or even during CCTA) may be useful in directing the treatment plan and delivering a functional stenosis of the anomalous artery [78]. Surgery is the method used to address coronary artery abnormalities. In particular, the risk of SCD is thought to be highest in cases of AOCA that originate from the incorrect sinus, have acutely angled take-off from the aorta, and have an abnormal path between the aorta and the pulmonary artery. Even in symptomatic patients, careful consideration should be given to surgically correcting such an abnormality. Moreover, it is not recommended to participate in high-intensity sports until a successful repair [81]. 

In the past, AOCP lacking an inter-arterial course—such as those with an aberrant right sinus origin of the circumflex artery were thought to have a low incidence of SCD [81]. Thus, there is no need for surgical repair or treatment if stress testing shows no arrhythmias, ischemia, or symptoms such as syncope, fainting, or dizziness in this setting. Guidelines recommend no restriction for competitive sport in asymptomatic individuals with AOCP that does not run between the large vessels, does not have a slit-like orifice with reduced lumen, and/or runs intramurally, provided that there is no inducible ischemia. However, these recommendations are subject to review following appropriate risk counseling [16]. Similarly, according to American guidelines, athletes with an abnormal RCA origin may resume sports participation following counseling as long as they exhibit no symptoms and pass a normal exercise stress test [82]. Conversely, if an athlete undergoes surgery and is asymptomatic with no indications of ischemia or arrhythmia on exercise stress testing, then participation in competitive sports should be limited for at least 3 months following surgical treatment of the lesion [82].

### 5.2. Coronary Artery Dissection

SCAD is caused by hematomas that form in the tunica media and consequently separates the intima or intima-medial complex from the underlying vessel, compress the actual lumen, and ultimately cause ischemia and myocardial infarction. Sex, hormone swings, pregnancy, underlying arteriopathies, heredity, and physical, mental, and emotional stressors are probably some of the variables that affect SCAD [83]. The true incidence of SCAD as an etiology of myocardial infarction in the general population is still unknown. In the general population, its incidence is around 1.1%, with 2.9% in those with MI and 4.2% in patients with unstable angina or postinfarction angina [84]. Moreover, a recent study found that SCAD is more frequent in female patients, with 23% of the cohort having multivessel SCAD [83]. However, SCAD incidence is underestimated as intravascular imaging is the only method available for correct diagnosing, and more than 70% of patients lack the traditional angiographic signs [84]. Even though it is a rare cause of exercise-induced CV events, it should be taken into account for any young individual who experiences acute coronary syndrome after chest injuries from sports or after intense activity.

Furthermore, observations and estimates of the incidence of exercise-induced SCAD have indicated noteworthy recurrence rates [85]. Therefore, intravascular imaging and coronary angiography play a critical role in the assessment of SCAD [83]. The Yip-Saw classification was created to help with the diagnostic pattern recognition of SCAD. It categorizes angiographic features into three types: Type 1 (a dual-lumen appearance caused by contrast penetrating the false lumen), Type 2 (a long, smooth stenosis), and Type 3 (a lesion that resembles a focal atherosclerotic disease). A precise diagnosis is essential for the appropriate management of SCAD, as a conservative strategy is frequently preferred over percutaneous coronary intervention [86]. 

American guidelines recommend that it is reasonable to restrict patients with prior SCAD to low-to-moderate dynamic and static demands, as SCAD can precipitate after exercise, even though there is not enough information to make firm recommendations regarding sports participation [87]. Similarly, European guidelines state that participation in competitive sports should be discouraged for predisposed athletes due to the increased rate of recurrence and the potential for severe cardiac injury or SCD. As a result, leisure activity should be advised and should be recommended individually for such patients [81].

### 5.3. Myocardial Bridge

MB is the term used to describe the partial or complete encasement of the coronary arteries by cardiac fibers. The artery’s intramyocardial part is known as a “tunneled artery,” and the muscle covering is called a “myocardial bridge”. Although the precise prevalence of MB is unknown, one in three persons most certainly have some kind of MB [88]. In general, it is regarded as a benign condition that is not linked to an increased risk of CV death, particularly when there are no other underlying conditions present. Systolic obliteration and milking of the intramyocardial portion of the epicardial coronary artery, however, may result in symptoms related to myocardial ischemia and, less frequently, an MI in the case of long (>25 mm) and deep (>2 mm) MB [89,90]. The inducible ischemia brought on by the MB’s compression is more noticeable during exercise. Sometimes, during imaging tests like coronary angiography or CCTA, performed to resolve the ambiguity of an aberrant workout ECG, MB might be incidentally found in athletes. As a gold standard tool, coronary angiography is typically required for confirmation. When there is a visible reduction of at least 70% in the minimal luminal diameter during systole and a persistent reduction of at least 35% in the minimal luminal diameter throughout mid-to late-diastole, there is a considerable “milking effect” (also known as systolic narrowing). Moreover, a variety of invasive approaches, such as intravascular ultrasound (IVUS), optical coherence tomography (OCT), doppler flow wire (DFW), and pressure wire methods, can be employed in the catheterization laboratory to perform a thorough evaluation of MB [88].

Managing is extremely difficult because there are no globally recognized guidelines for treating MB. When treating MB, medical management, which may involve the use of ivabradine, beta-blockers, or calcium channel blockers, should be the first line of treatment. Revascularization should be explored if symptoms do not improve after receiving the most aggressive medical treatment. This usually entails cardiac surgery, such as coronary artery bypass grafting (CABG) or supra-arterial myotomy, also referred to as “unroofing”. While coronary stenting is normally not recommended, there is evidence that a high radial strength drug-eluting stent platform may be helpful in certain situations [88].

Competitive sports are not advised for people with MB and chronic ischemia or complicated cardiac arrhythmias during maximal exercise stress testing, according to American and European standards. On the other hand, in asymptomatic patients with MB who do not exhibit ventricular arrhythmia or inducible ischemia after maximal exercise testing, sports participation, both competitive and recreational, should be taken into consideration [16,87].

Moreover, according to the American Heart Association, athletes who have had their MB surgically removed or stented should be limited to low-intensity sports for 6 months following the treatment. These athletes are eligible to compete in any sport if they do not show any signs of ischemia later on [87].

## 6. Management of Athletes with Suspected Coronary Artery Disease

### 6.1. Evaluation of Athletes at Risk

Compared to non-athletes, athletes with myocardial ischemia often have a lack of symptoms despite subclinical CAD due to the greater coronary reserve. Indeed, because of exercise-related ischemia preconditioning and coronary collateralization, elite athletes may have CAD without any symptoms [16]. However, a decrease in exercise capacity that is nevertheless greater than that of the non-athletic population, tachycardia, angina with extremely high exercise workloads, and unexpected mortality may be verified in some athletes with CAD. Previous research by Braber et al. revealed that up to 19% of 318 middle-aged male athletes who received a sports medical evaluation without abnormalities had a CACS of ≥100 and/or ≥50% coronary artery stenosis [33]. According to these results, a significant number of athletes who do not exhibit symptoms may have subclinical atherosclerosis, which may go unnoticed during sports medical examinations and put them at risk of cardiac issues brought on by exertion.

Thus, screening is crucial, particularly for competitive athletes. An exercise test, physical examination, ECG, and medical history are usually included in a sports medical evaluation. Preparticipation screening techniques have been suggested to reduce the risk of SCD associated with sports, although they have not been thoroughly tested in elite athletes. American Heart Association 14-element guidelines, the FRS, and an ECG were among the preparticipation screening tools used on 798 elite athletes as part of the Master Athlete Screening Study (MASS) [91]. The strongest predictor of CAD in elite athletes was a high FRS, which, as recommended, should be included in the preparticipation screening procedure. However, the authors speculate that in symptomatic elites, those with a family history of SCD and intermediate-high FRS also exercise stress testing may be indicated [91].

Nevertheless, other methods have been investigated with the aim of predicting CV risk in athletes. However, the traditional risk variables are not significantly increased by more recent predictive metrics like carotid intima-media thickness (IMT) and high-sensitive C-reactive protein [16]. While implementing CAC screening programs for middle-aged and older athletes is not currently advised, one could argue that, given the continuous debate about better CV screening of athletes, this may become an option in the future [16]. Although newer studies have cast doubt on this idea [41], it is plausible that the presence of only elevated CAC among elite endurance athletes may very well represent a clinically benign phenotype.

The ESC guidelines recommend using the SCORE system, which takes into account factors such as age, sex, smoking status, LDL-cholesterol, and BP, to predict the risk of a fatal CV event in the next ten years, to identify athletes with risk factors for CAD and subclinical chronic coronary syndrome (CCS) during preparticipation screening [16]. A growing proportion of people with asymptomatic CCS, including competitive elite athletes, can be identified mainly due to the growing use of cardiac imaging modalities in addition to the SCORE risk assessment. Elite athletes with a SCORE < 5% are deemed low risk. If they are asymptomatic and engage in PA, they are free to continue participating in sports at any intensity. On the other hand, individuals who have SCORE ≥ 5% are recommended to undergo further testing, a high-intensity ECG exercise test, or an equivalent stress test [16,92]. However, the last European guidelines on CV disease prevention introduced the second version of SCORE (SCORE2), which intended to assess the 10-year risk of both fatal and non-fatal CV events in apparently healthy individuals [1]. In addition, age-specific thresholds are used to classify individuals into three risk categories: low-to-moderate risk (<2.5% if <50 years old or <5.0% if aged 50–69 years), high risk (2.5 to <7.5% if <50 years or 5.0 to <10% if 50–69 years) and very high risk (≥7.5% if <50 years or ≥10% if 50–69 years). Moreover, a customized version for older people (SCORE2-OP) was released, and both SCORE2 and SCORE2-OP were calibrated to four clusters of countries (low, moderate, high, and very high CV disease risk) that are categorized according to national CV mortality rates [1].

Nonetheless, most of the research used CCTA to assess patients who were at risk of CAD. Several worldwide guidelines recommend CCTA as the first test for screening general individuals with suspected CAD and low-to-intermediate pre-test likelihood [93]. Moreover, it is a commonly accessible test. As previously described, the CACS is a highly sensitive measure in case of the existence of >50% angiographic stenosis in both men and women. A CACS of zero, however, does not have the same high negative predictive value in symptomatic individuals as it does in asymptomatic people. Nevertheless, overall plaque burden as determined by CAC can offer more details that may aid in risk assessment [68,94]. Furthermore, high-risk plaque (with thin fibrous cap, big plaque volume, necrotic core, and spotty calcification) and anomalous coronary arteries can both be detected by CCTA [68]. However, there are several limitations to CCTA. Numerous studies contrasting functional testing with CCTA have shown that CCTA leads to a much higher rate of revascularization and significantly higher utilization of invasive coronary angiography as compared to functional testing [95]. Because athletes’ plaque composition is more constant than that of the general population, CAC scores could also overestimate CV risk. Additionally, information about coronary flow and reserve cannot be obtained from anatomical coronary imaging. Thus, maximal exercise testing would probably be beneficial for individuals with considerably elevated CACS in order to assess LV function and inducible ischemia or electrical instability [95].

The most common examination for athletes with suspected or confirmed CAD is the exercise test. Its goals are to identify potential stress-induced myocardial ischemia, assess overall exercise capacity, measure BP and heart rate responses, identify exercise-induced arrhythmias, and track the evolution of symptoms. However, the low sensitivity, intermediate specificity, and frequently unclear test results for the detection of morphological CAD in individuals without known CAD compromise the diagnostic accuracy [93]. In low-risk and asymptomatic patients, their specificity for myocardial ischemia is significantly lower than that of other functional tests [16]. 

Therefore, functional stress testing such as stress echocardiography, positron emission tomography (PET)/single-photon emission computed tomography (SPECT), adenosine or dobutamine stress CMR, should be performed on patients with borderline exercise test or doubtful test [16]. Exercise or pharmacologic stress during TTE can identify ischemia based on the appearance of new regional wall motion abnormalities or a decrease in previously existing wall motion abnormalities. Severe obstructive CAD, such as left main stenosis or multivessel CAD, is suggested if the global LVEF declines and/or the LV end-systolic volume rises. Additionally, stress echocardiography can offer information on hemodynamics during exercise [96]. Hemodynamic stress echocardiography studies can yield parameters of diastolic function, systolic pulmonary artery pressure responses, and valvular heart disease. Nevertheless, myocardial ischemia may go undetected with imaging during the recovery phase because athletes have a quick heart rate recovery following exercise. Because images may be obtained at peak exercise, bicycle stress echocardiography may be a more appropriate diagnostic method [96]. 

SPECT is another useful instrument. A well-researched, widely accessible method for assessing individuals with suspected or confirmed CAD is pharmacologic or exercise stress using gated (ECG-triggered) SPECT. In this setting, coronary artery ischemia is thought to be indicated by cardiac segments that exhibit intact myocardial perfusion at rest but reduced myocardial perfusion during stress [97]. Moreover, functional testing with SPECT produces comparable results to CMR in cases of suspected CAD [98]. However, some of the most significant limitations of these methods are radiation, costs, and the possibility of false positives in some situations. 

Currently, CMR is the gold standard for non-invasive evaluation of the left and right ventricles of the heart, cardiac mass, valve regurgitations, and, ultimately, infarct size. Measuring flow, as well as evaluating tissue properties and CV architecture are key elements when using CMR [99]. Furthermore, extracellular contrast agents have become more beneficial for both ischemia and non-ischemic scar visualization. In addition, CMR can accurately diagnose Takotsubo syndrome, myocarditis, and old versus recent myocardial infarction, as well as congenital high-risk aberrant origin of the coronary arteries [100]. However, due to its expensive cost and lack of widespread availability, its use is irregular. 

Even though functional stress imaging is crucial for evaluating CCS, experts cannot agree on which approach is better for athletes who are at risk of CAD. Finally, deciding when to administer cardiac injury indicators is equally difficult. Due to the possibility of elevated cardiac troponin (cTn) I and T levels, which are serologic indicators of myocardial injury associated with severe exercise, the diagnosis of myocardial injury in athletes is also more complicated [101]. Serum cTn must be measured in cases of post-exercise complaints such as palpitations, syncope, inappropriate breathing difficulties, and chest discomfort that is not immediately related to neuro-cardiogenic mechanisms (such as musculoskeletal damage, dermatologic complaints, dehydration, malnutrition, and thermal injury). In some cases, with low cardiac risk but very high cTn, additional cTn I or other muscle injury indicators measurement may be useful. Troponin kinetics can be effectively determined by repeating samples, which can help differentiate between cTn rise associated with exercise and other causes in stable persons [102].

A correct evaluation using a combination of different diagnostic methods can help in choosing the most suitable management plan for a single athlete with suspected CAD.

### 6.2. Management of Athletes with Coronary Artery Disease

The number of athletes with apparent subclinical CAD disease has increased due to the increased availability and interest in CAC assessment. Even though the true risk profile of these athletes may differ slightly from that of the general population, it is generally accepted that strategies aimed at modifying established and emerging atherosclerotic risk factors with statin and antiplatelet therapy are more appropriate when confronting them. Nonetheless, thoughtful and collaborative decision-making sessions concerning the patient’s specific training and competition objectives are crucial. Activity suggestions should be customized for each individual based on the type of sport and the intensity of the activity, adhering to European and/or American guidelines. However, aggressive management of atherosclerotic risk factors is recommended for both CAD-asymptomatic persons and those at risk if screening reveals CAD in these individuals [16]. 

In the era of digitalization, wearable device monitoring of elevated BP or heart rate is becoming more common, and athletes with clinical or subclinical CAD may be the intended audience for this innovative telemonitoring approach. Different wearable devices and applications are available to provide measurements of heart rhythm and BP monitoring, and some of them include Scanadu Scout, Apple’s iWatch, smartphone accelerometers, and smartphone-based cuffless BP, as well as Google Glass wearable gadgets and Masimo Personal Health, the probe-based smartphone application [103]. Wearable BP monitoring devices providing a vast amount of BP data in different conditions and simultaneous monitoring of environmental conditions will allow more accurate diagnosis of phenotypes that have a negative impact on cardiovascular prognosis, such as masked hypertension and pathological BP variability [104]. However, it will need a cooperative, interdisciplinary strategy involving patients, physicians, scientists, legislators, and business executives to change the digital health environment and enable wearable technology to realize its full therapeutic potential [105].

Further assessment with coronary angiography is required for elite athletes who have proven CAD and inducible ischemia on maximal-effort stress testing, and sports limitations may also be applicable. In general, athletes can engage in any intensity exercise program provided they have low-risk coronary lesions, no symptoms, preserved LVEF, and no arrhythmias during maximal-effort exercise testing (though individual restrictions may apply, such as competing in triathlons) [16].

Conversely, people with long-standing CCS who have preserved LVEF, do not exhibit any abnormalities on a maximal activity test, or both may be regarded as low risk of an exercise-related adverse event. On an individual basis, these people can participate in any competitive sport. Athletes, on the other hand, may develop high-risk coronary lesions, which are characterized as >90% lesion in the coronary vessel, multivessel disease (at least 2 lesions with >50% stenosis), or >50% on left main or proximal left anterior descending. These lesions characterized by coronary angiography should be associated with a history of proven ischemia or be classified as hemodynamically significant if their FFR or iFR is less than 0.8 or 0.89, respectively [16].

It is recommended that individuals with high-risk coronary lesions have revascularization before being evaluated for sports. The highest level of exercise advised is moderate leisure sport and participation in individually recommended low-intensity skill sports, with a restriction on intense competitive athletics if revascularization is not possible or if the athlete continues to exhibit ischemia despite optimal medical therapy. After revascularization, if a patient has a normal maximal exercise test (no ischemia or arrhythmia), a normal LVEF, and no symptoms, they can resume full-intensity sports after 3–6 months [16].

The American College of Cardiology (ACC)/AHA guidelines (Table 1) offer comparable recommendations. However, they omit guidance on CV risk stratification in athletes who are asymptomatic and have CV risk factors but no known CAD [87]. Furthermore, before beginning high-intensity competitive sports, people who have had an acute coronary syndrome (ACS), heart surgery, or percutaneous intervention should be referred to an early cardiac rehabilitation program as soon as they are discharged. This program should last for 8 to 12 weeks following the cardiac event and include a thorough evaluation using cardiopulmonary exercise testing [16]. On the other hand, ACC/AHA guidelines state that it is reasonable to forbid patients with clinically manifest ACS from engaging in competitive sports for a minimum of three months following an MI or coronary revascularization procedure or if their myocardial ischemia symptoms are becoming worse or more frequent [87].

Even with the sports suggestion, treating CAD in elite athletes in everyday practice remains difficult. Patients can receive appropriate medical therapy or an invasive method if they have stable coronary disease and mild to severe ischemia. In fact, in the ISCHEMIA (International Study of Comparative Health Effectiveness with Medical and Invasive Approaches) trial [106], individuals with stable CAD treated with a conservative first medical therapy approach or an invasive strategy of angiography and coronary revascularization had equal 3-year risks of ischemic events. Furthermore, there was a lower risk of CV mortality and a higher risk of non-CV mortality with an initial invasive strategy compared to an initial conservative strategy during the extended follow-up analysis (5.7 years). However, there was no difference in all-cause mortality [107]. 

Comparably, percutaneous coronary intervention (PCI) and optimal medical therapy (OMT) or OMT did not show differences in terms of all-cause mortality in the COURAGE trial. There has not been much success in identifying subgroups of COURAGE patients that may have a survival benefit following an initial approach of PCI with OMT [108,109]. The previously mentioned rules should be observed by athletes who intend to pursue higher athletic intensities and for which the ISCHEMIA data are not applicable. In fact, revascularization may be preferred as a treatment for myocardial ischemia in athletes who engage in greater-intensity exercises, combined power and endurance sports, or high-endurance sports [16]. If PCI is the preferred treatment, it is imperative to prevent stent under-expansion, as this could increase the risk of stent thrombosis, as both clinicians and athletes may choose a shorter DAPT period. Moreover, complete revascularization, when feasible, can allow for a reduction in the number of drugs and adverse effects. 

As previously shown, guidelines advise revascularization in elite athletes in order to reduce the ischemic substrate, even though there is little evidence that this may lessen the risk of myocardial infarction or SCD [1,16]. However, a recent case series showed that among 798 screened athletes, 6 asymptomatic elite athletes with ischemia on ECG exercise stress testing had their coronary anatomy defined either by cardiac computed tomography or coronary angiography and treated with optimal medical therapy, where only 1 underwent PCI. They demonstrated a lack of clinical events after 4.3 years of follow-up, whether or not revascularization was performed [109]. However, athletes may benefit greatly from PCI even though it might not prevent MACE. In a recent investigation, coronary pressure-flow measurements were made during exercise on a supine ergometer in 21 patients with stable coronary disease and single-vessel coronary stenosis. PCI quickly restored the ability of coronary flow and pressure and improved coronary, microvascular, and systemic hemodynamic responses. Moreover, they showed that PCI restores the coronary artery to its primary function as a conduit and the ability of the microcirculation to gradually vasodilate during exercise, by instantly eliminating stenosis resistance [110]. In any case, athletes with stable CAD who have undergone PCI should be referred to a cardiac rehabilitation program to maximize their medication and lifestyle changes while tracking their return to their prior activity routine [16].

Additionally, doing a functional coronary examination could be another solution for the management of athletes with CAD. Because these measurements were developed in a non-athletic community, the traditional angiographic functional measurement cut-offs (FFR ≤ 0.80/iFR ≤ 0.89) would not be as generalizable in extremely athletic populations [111,112]. Therefore, the importance of persistent ischemia during endurance exercise and the precise definition of a comparable quantity of ischemia on invasive or non-invasive tests remain uncertain. Indeed, it is also critical to recognize that CRF was not taken into account in any of the studies that tested these thresholds, including Fractional Flow Reserve Versus Angiography for Multivessel Evaluation (FAME) and Fractional Flow Reserve Versus Angiography for Multivessel Evaluation 2 (FAME 2) [111,112]. It is possible that the FFR cut-off values now in use are inappropriate for evaluating a flow-limiting lesion at the highest levels of physiological stress experienced by elite athletes. Do athletes play by different rules when it comes to revascularization is still unknown [109].

Another concern is whether there is any tolerated ischemia for high-intensity athletes. Furthermore, there are disagreements on the necessity of revascularization in middle-aged athletes who are asymptomatic and have obstructive CAD and visible ischemia. However, intravascular imaging and plaque assessment may be crucial in the future when it comes to elite endurance athletes and PCI. 

Despite the still unanswered questions, for all elite athletes with obstructive CAD, decisions on evaluation, medication therapy, and revascularization must be made on an individual basis using a shared decision-making approach, not forgetting that aggressive risk factor modification is crucial [109].

### 6.3. Gaps in Evidence and Future Directions

There are still many gaps in the research, even though numerous studies have attempted to assess CAD in athletes. In many trials, the most often utilized CCTA parameter is CAC. Nevertheless, novel metrics above the calcium score have surfaced as auspicious approaches to assessing CV risk. The more promising epicardial adipose tissue volume is linked to increased MACE, high-risk plaque development, and the progression of atherosclerosis. The others include liver fat (a sign of cardio-metabolic health and the development of atherosclerosis), myocardial scar (an indicator of the worst prognosis in patients with myocardial infarction), and aortic calcium (able to improve CV risk stratification beyond CACS) [68]. These new CCTA applications will eventually enable improved prognostic stratification and early identification of patients at risk of unfavorable clinical outcomes and severe atherosclerosis.

Moreover, studies suggest that endurance training reduces the occurrence of ischemia events independently of CACS, indicating that more research is necessary to fully understand the dynamics of atherosclerosis. This is because endurance athletes may have larger coronary arteries and higher vasodilatory capability [41,113,114,115]. Indeed, extensive prospective longitudinal studies with elite athletes are necessary to explore the underlying causes and the importance of coronary plaques, myocardial fibrosis, and CAC in athletes. It is unclear what processes underlie the results of increased coronary atherosclerosis in athletes. This question cannot be answered by the studies and literature now in existence. Therefore, it will probably be essential to conduct animal research as well as physiological clinical trials to determine which molecular pathways are responsible for these data. In this setting, more research is warranted to fully understand the possible significance of inflammation in these findings.

Furthermore, more study is required to examine the qualitative and quantitative burden of disease based on gender and race and in non-endurance athletes.

Management of these patients remains challenging as well. Indeed, further research is required to determine the role of PCI, as well as precise FFR and iFR cut-offs that accurately represent the risk/benefit ratio of PCI in competitive athletes with CAD. Perhaps combining intravascular ultrasonography (IVUS), optical coherence tomography (OCT), or near-infrared spectroscopy (NIRS) may help identify individuals who may need PCI based on plaque vulnerability [116]. Future investigations should concentrate on clarifying the molecular mechanisms underlying the development and advancement of coronary plaque in athletes, investigating the long-term impacts of PA on plaque stability, susceptibility, and associated events, and assessing the effectiveness of specific therapies meant to reduce CV risk among elite athletes.

## 7. Conclusions

PA has numerous widely recognized health benefits, but it should be noted that it does not make athletes immune from heart diseases. The intricacy of atherosclerosis in this group is highlighted by data from the literature, which also raises important issues about the relationship between PA, CV risk, and plaque morphology. The present review summarizes current evidence about the intriguing dynamics of CAD among athletes, offering insights into its prevalence, underlying mechanism, clinical and prognostic implications, and challenges in its evaluation and management. 

Elevated levels of PA are linked to elevated CACS values and atherosclerotic load, mostly defined by calcified plaques that were previously thought to be innocuous. It is crucial to keep in mind, though, that calcified plaque is not always associated with a decreased risk of CV events in athletes. Instead, additional characteristics of the plaque and CCTA indicators may be more significant in determining the stability and vulnerability of the plaque.

Moreover, subclinical cardiac issues are often underdiagnosed and undertreated among endurance athletes. Therefore, even with lesser occurrences, it is crucial to emphasize that, in addition to the atherosclerotic process, athletes may also have other coronary abnormalities, such as AOCA, MB, and SCAD, which should be considered and ruled out.

Despite the unresolved challenges, choices about assessment, pharmacological therapy, or revascularization for all elite athletes with obstructive CAD must be decided individually using a shared decision-making method.

It will be a difficult task to provide clarity to this clinical conundrum that should be resolved by future large-scale investigations.

## Figures and Tables

**Table 1:** A comparison of European and American guidelines regarding CAD athletes’ management and eligibility for sporting competition.

Guidelines ^1^	Settings	Competitive Sports Allowed if	Competitive Sports Not Recommended if
ESC	Clinically manifest CAD in the setting of CCS	- Preserved LVEF, no abnormalities on a maximal exercise test or functional imaging test, no high-risk coronary lesions	- High-risk coronary lesions ^2^ not eligible for revascularization - LVEF ≤ 50% - Inducible myocardial ischemia or NSVT/frequent PVC
	Asymptomatic CAD ^3^	- Disease progression during serial evaluations - Positive maximal exercise test or functional imaging test	- No inducible myocardial ischemia on functional imaging or conventional exercise stress
	Acute coronary syndrome	- After 8–12 weeks of cardiac rehabilitation and 3–6 months of structured outpatient exercise programs - Normal exercise test with 12-lead ECG recording or CPET before returning to sport competition	- First 3–6 months after the event - Persistent inducible myocardial ischemia or Symptoms after successful revascularization
	Post-revascularization	- After 3–6 months, if negative stress testing, normal LVEF, and no symptoms	- Ischemia that cannot be treated despite adequate therapy
ACC/AHA	Clinically manifest CAD in the setting of CCS	- LVEF > 50%, asymptomatic, no inducible ischemia or electrical instability	- Patients non-fulfilling 1 or more criteria: LVEF > 50%, asymptomatic, no inducible ischemia or electrical instability
	Asymptomatic CAD ^4^	X	X
	Acute coronary syndrome	- After 3 months of the event - No symptoms and no inducible myocardial ischemia	-Increasing frequency or worsening symptoms of myocardial ischemia - First 120 days after ACS
	Post-revascularization	- After 3 months following PCI - No symptoms and no inducible myocardial ischemia	-Increasing frequency or worsening symptoms of myocardial ischemia - First 120 days after ACS

^1^ Despite the generally accepted recommendations, both guidelines [16,87] emphasize that participation in all types of exercise, including competitive sports, should be based on individual assessment. ^2^ Lesions > 90% in the coronary vessel, multivessel disease (at least 2 lesions with >50% stenosis), or >50% on left main or proximal left anterior descending. These lesions characterized on coronary angiography should be associated with a history of proven ischemia or be classified as hemodynamically significant if FFR < 0.80 or iFR < 0.89. ^3^ Elderly patients (>65 years), elite athletes may participate in competitive sports if asymptomatic and at low or moderate cardiovascular risk (class IIB, Level C). ^4^ No recommendations for asymptomatic athletes with suspected CAD. ACC = American College of Cardiology, AHA = American Heart Association, ACS = acute coronary syndrome, CAD = coronary artery disease, CCS = chronic coronary syndrome, CPET = cardiac, pulmonary exercise testing, ECG = electrocardiogram, ESC = European Society of Cardiology, LVEF = left ventricular ejection fraction, NSVT = non-sustained ventricular tachycardia, PCI = percutaneous coronary intervention, PVC = premature ventricular contractions.
